# Systemic TNF blockade does not modulate synovial expression of the pro-inflammatory mediator HMGB1 in rheumatoid arthritis patients – a prospective clinical study

**DOI:** 10.1186/ar2387

**Published:** 2008-03-17

**Authors:** Erik Sundberg, Cecilia Grundtman, Erik af Klint, Johan Lindberg, Sofia Ernestam, Ann-Kristin Ulfgren, Helena Erlandsson Harris, Ulf Andersson

**Affiliations:** 1 Department of Woman and Child Health, Pediatric Rheumatology Research Unit, Karolinska Institutet/Karolinska University Hospital, Stockholm, Sweden; 2 Department of Medicine, Rheumatology Unit, Karolinska Institutet/Karolinska University Hospital, Stockholm, Sweden; 3 Department of Biotechnology, AlbaNova University Center, Royal Institute of Technology, Stockholm, Sweden

## Abstract

**Introduction:**

High-mobility group box chromosomal protein 1 (HMGB1) has recently been identified as an endogenous mediator of arthritis. TNF and IL-1β, pivotal cytokines in arthritis pathogenesis, both have the ability to induce the release of HMGB1 from myeloid and dendritic cells. It was, therefore, decided to investigate whether treatment based on TNF blockade in rheumatoid arthritis (RA) affects the expression of synovial HMGB1.

**Methods:**

Repeated arthroscopy-guided sampling of synovial tissue was performed in nine patients with RA before and nine weeks after initiation of anti-TNF mAb (infliximab) therapy. Synovial biopsy specimens were analysed for HMGB1 protein by immunohistochemical staining and for HMGB1 mRNA expression by real-time reverse transcriptase PCR (RT-PCR). Statistical evaluations were based on Wilcoxon's signed rank tests or Spearman rank sum tests.

**Results:**

Aberrant, extranuclear HMGB1 and constitutive nuclear HMGB1 expression, with histological signs of inflammation, were evident in all biopsies obtained before infliximab therapy. Signs of inflammation were still evident in the second biopsies obtained nine weeks after initiation of infliximab therapy. The cytoplasmic and extracellular expression of HMGB1 decreased in five patients, remained unchanged in one patient and increased in three patients, making the overall change in HMGB1 protein expression not significant. No correlation between the clinical response, as measured by disease activity score calculated for 28 joints (DAS28) or the American College of Rheumatology response criteria (ACR 20, 50, and 70), and the direction of change of HMGB1 expression in individual patients could be discerned. In addition, infliximab therapy did not alter HMGB1 mRNA synthesis.

**Conclusion:**

Pro-inflammatory HMGB1 expression during rheumatoid synovitis was not consistently influenced by TNF-blocking therapy with infliximab. This suggests that TNF is not the main inducer of extranuclear HMGB1 during synovitis and that HMGB1 may represent a TNF-independent molecule that could be considered as a possible target for future therapeutic intervention in RA.

## Introduction

Rheumatoid arthritis (RA) is an autoimmune disease characterised by chronic polyarticular inflammation leading to the destruction of cartilage and subchondral bone. The pathogenesis of RA is complex, involving a wide range of endogenous pro-inflammatory molecules including cytokines. Certain mediators, with TNF as one causative molecule, can be successfully targeted in the treatment of chronic arthritis. TNF-blocking therapy has been shown to dramatically reduce inflammation and tissue destruction in many patients with RA [1-3]. However, it is also evident that anti-TNF therapy is not effective in all patients and that many responders still present residual signs of active disease. In order to improve the treatment of chronic arthritis, a further search for additional potential target molecules that act independently of TNF is highly warranted.

Recent findings have suggested that the high-mobility group box chromosomal protein 1 (HMGB1) might be an important molecule in the pathogenesis of arthritis [4-10]. Intranuclear HMGB1 binds DNA and regulates transcription. In addition, HMGB1 may be extracellularly translocated, thereby acting as an inflammatory mediator of tissue invasion and tissue repair [11-18]. HMGB1 may either be actively secreted from a wide number of cell types following stimulation with inflammatory mediators, including TNF, IL-1β, IFN-γ and multiple toll-like receptor (TLR) ligands [15,19-23], or be passively released from dying nucleated cells [12,13]. The extracellular effects of HMGB1 are mediated via multiple receptors including the receptor for advanced glycated end-products (RAGE), some members of the TLR family and other as yet unidentified pathways [17,24-26]. Increased levels of HMGB1 are evident in the synovial fluid of patients with RA and HMGB1 has been shown to be abundantly expressed in an aberrant fashion in rheumatoid synovial tissue [4,6]. Serum levels of HMGB1 are also elevated in patients with RA and correlate with disease activity [27]. In addition, intra-articular injections of HMGB1 trigger destructive arthritis in naive mice [5].

Different modes of HMGB1-blocking therapy, including neutralising antibodies, antagonistic truncated HMGB1, soluable RAGE (sRAGE), thrombomodulin or nuclear HMGB1 sequestration, have been successfully applied in studies of experimental arthritides and sepsis [15,28-33]. It was recently reported that gold salts interfere with the intracellular transport mechanisms of HMGB1 and inhibit its release [34]. Oxaliplatin is an antineoplastic platinum-based compound that generates DNA adducts that strongly bind HMGB1. Therefore, gold salts and oxaliplatin share the capacity to inhibit nuclear HMGB1 release via different mechanisms. Short-term oxaliplatin treatment in collagen type-II-induced arthritis was recently studied in mice and beneficial therapeutic effects coinciding with nuclear HMGB1 retention were noted [35]. Once released, HMGB1 might generate a positive feedback loop and induce production of several pro-inflammatory cytokines such as IL-6, IL-1β and TNF by macrophages and dendritic cells, thereby sustaining prolonged inflammation [16,36].

In this pilot study the aim was to analyse to what extent extranuclear HMGB1 expression depends on and relates to TNF levels in RA, as previous studies have indicated that TNF can induce HMGB1 release. Synovial biopsy specimens from patients with RA were collected by arthroscopy before and during therapy with TNF-specific mAb (infliximab) and the levels of synovial expression of HMGB1 protein and mRNA were evaluated.

The main findings were that synovial HMGB1 protein and mRNA expression did not change in any consistent manner after nine weeks of infliximab treatment.

## Materials and methods

### Patients, clinical assessment and therapy

Nine patients (seven females and two males) with RA diagnosed according to the revised American College of Rheumatology (ACR) criteria [37] and active knee arthritis were enrolled in a prospective clinical study. Informed consent was obtained from all patients and the study was approved by the local ethical committee at Karolinska University Hospital, Stockholm, Sweden.

The median age of patients was 57 years (range 25 to 69 years) and the median disease duration was six years (range 0.6 to 18 years). The median duration of the current episode of arthritis in the knee was 17.5 days (range 3 to 365 days; no data for one patient). In all patients the disease activity score calculated for 28 joints (DAS28) and the ACR response criteria (ACR20, 50, and 70) were assessed at multiple time points before and during therapy. The median DAS28 at inclusion was 5.95 despite treatment with methotrexate (7.5 to 17.5 mg/week). Methotrexate doses were stable during the study and for at least one month before the first arthroscopy. Four patients received prednisone at stable doses (5 to 7.5 mg/day) during the study period. One patient received cyclosporine (150 mg/day) before and during the study period.

Infliximab (Centocor BV, Leiden, The Netherlands) was given as three intravenous infusions of 3 mg/kg in accordance with the recommended standard-treatment protocol, with the first infusion given 1 to 21 days after the first arthroscopy and the subsequent infusions given two and six weeks later. The results of synovial expression of IL-15 in response to infliximab therapy using the same cohort of patients with RA have previously been published [38-40].

### Synovial biopsies

Knee arthroscopy with multiple biopsies of synovial tissue of the knee joint was performed in all patients 1 to 21 days before the first infliximab infusion. A second arthroscopy was performed at 8 to 10 weeks (median nine weeks) after the first infusion, a time point when infliximab therapy is well established and a clinical response can be evaluated [1].

All arthroscopies were performed by the same experienced physician (EK). During the first arthroscopy, multiple synovial tissue biopsies were taken from areas with signs of maximum macroscopic inflammation, from the cartilage-pannus junction and from synovial villi. Each biopsy site was documented photographically and mapped, allowing for follow-up biopsies to be taken from the same areas. The biopsies were snap-frozen within two minutes in liquid isopentane and stored at -70°C until sectioned. Serial cryostat sections (7 μm) were fixed for 20 minutes with 2% (v/v) formaldehyde and stored at -70°C. Several biopsies were taken to secure sufficient material for subsequent analyses. For each of the nine patients the best biopsy pair with respect to morphology was selected for subsequent immunohistochemical stainings. The staining was always performed for samples taken before and after infliximab treatment allowing for a pairwise comparison.

### Immunohistochemistry

For indirect immunohistochemistry evaluation, tissue sections were blocked for non-specific binding with H_2_O_2 _and NaN_3 _for one hour, with normal goat serum (X0907, DAKO, Glostrup, Denmark) for 15 minutes and with an Avidin/Biotin blocking kit (SP-2001, Vector Laboratories, Burlingame, CA USA) according to the manufacturer's instructions. Saponin (0.1% w/v in HEPES pH 7.2) was added throughout the staining protocol. An HMGB1-specific polyclonal peptide-affinity purified rabbit antibody (PharMingen 556528, San Diego, CA, USA) was used as the primary antibody at a final concentration of 0.5 μg/ml. A non-specific rabbit antibody (X0902, DAKO, Glostrup, Denmark) was used as the control. To identify blood vessels, a human endothelium-specific mAb (anti-EN4, anti-human CD31 from Sanbio, Bio-Zac, Uden, The Netherlands) was used. As the negative control for CD31, an irrelevant mouse IgG_1 _mAb (DAKO, Glostrup, Denmark) was also used.

Sections were incubated with primary antibodies overnight. The secondary antibody was a biotinylated goat anti-rabbit IgG (BA-1000, Vector Laboratories, Burlingame, CA, USA) diluted to 1:800 for HMGB1 detection, and a biotin goat anti-mouse IgG_1 _(DAKO, Glostrup, Denmark) was used for CD31 detection. Extra-avidin peroxidase (EAP) (Sigma, St. Louis, MO, USA) with diaminobenzidine (DAB) (Vector Laboratories, Burlingame, CA, USA) as substrate were used for visualisation and sections were then counterstained with haematoxylin, washed, dried and mounted with buffered glycerol.

Two evaluators (ES and CG), blinded to the order of the sections, performed a semi-quantitative analysis of the expression of HMGB1. Scoring for each section was evaluated using a 0 to 4 scale with increments of 0.5. Index 0 corresponded to no HMGB1 expression and 4 to the highest degree of HMGB1 protein expression. Separate analyses were performed for lining layers, vessels and cellular infiltrates in each of the sections. Nuclear, cytoplasmic and extracellular expression of HMGB1 was also recorded from each tissue compartment.

### Real-time RT-PCR

Due to a shortage of biopsy material, mRNA analysis was only possible in six of the nine included patients. For first-strand synthesis of each biopsy, 1 μg total RNA was mixed with 2 μl 20 TVN primer (4 μg/μl, Operon Biotechnology Inc. Huntsville, AL USA). The primer sequences used were: for β-actin, forward CCTTCGTGCCCCCCC and reverse GGAGACCAAAAGCCTTCATACATC; and for HMGB1, forward ATTGGTGATGTTGCGAAGAA and reverse GATCCACAGCAACTCCAGAA. The volume was adjusted to 15.5 μl using RNase-free water, and the mixture was then incubated for 10 minutes at 70°C to denature the total RNA. The sample was then incubated for two minutes on ice to allow the primers to anneal and then spun briefly.

A 12.5 μl cDNA synthesis mixture, consisting of 6 μl 5× first-strand buffer, 3 μl 0.1 M dithiothreitol (DTT), 2 μl Superscript III (Invitrogen Corporation, Carlsbad, CA, USA), and 1.5 μl 10 mM deoxinucleoside triophosfate (dNTP) mix (Amersham Biosciences, Piscataway, NJ, USA), was added to the sample. The whole mixture was incubated at 46°C for first-strand synthesis. After one hour the temperature was increased to 70°C for 15 minutes to terminate the reaction. 2 U RNase H (Invitrogen Corporation, Carlsbad, CA, USA) was then added to degrade the RNA. After RNase treatment the temperature was increased to 70°C to inactivate RNases. All samples were then diluted with RNase-free water to a final volume of 200 μl.

Real-time RT-PCR was performed using the iCycler system from Bio-Rad Laboratories Inc. Hercules, CA, USA. Each reaction was performed with a 3.0 μl template, 12.5 μl iQSYBR Green Supermix (Bio-Rad Laboratories Inc. Hercules, CA, USA), 300 nM primer and water to adjust the final volume to 25 μl. The PCR amplification steps were applied in the following conditions: three minutes at 95°C, 40 cycles of 20 seconds at 94°C, 30 seconds at 60°C and one minute at 72°C. This was followed by melt curve analysis to ensure specific amplification. The signal was calculated in all experiments using 'PCR baseline subtracted relative flourescent unit (RFU)'. All primers were designed using Primer3 and amplicons were designed to span exon-exon junctions to minimise contamination of genomic DNA [41]. Primer sequence information is available in the supplemental material. Relative expression between samples was calculated using the ΔΔCt method and all samples were analysed in triplicate[42]. β-actin was used as a reference housekeeping gene.

### Statistical analysis

Wilcoxon's signed-rank test was used for the analysis of matched pairs for protein data. as well as for the analysis of mRNA data for the whole group. Spearman rank sum test was utilised to statistically compare the degree of correlation between the two persons evaluating HMGB1 protein expression by immunohistochemistry. p < 0.05 was considered to be statistically significant.

## Results

### Clinical response and CD marker changes following infliximab treatment

Patients were assessed for disease activity at baseline and after three months using individual DAS28 and ACR scores (Table 1). These data have been previously published [38]. Median DAS28 scores decreased from 5.95 to 4.41 (p < 0.01), the median tender joint count from 10 to 3 (p < 0.05), the median swollen joint count from 14 to 2 (p < 0.01) and the median serum C-reactive protein level decreased from 34 mg/L to 19 mg/L (p = 0.08). Two patients fulfilled ACR70, one patient fulfilled ACR50, three patients fulfilled ACR20 and three patients were non-responders according to the ACR criteria.

**Table 1:** Clinical assessment, response to infliximab treatment and HMGB1 expression.

Pat	W	Sex	Age (years)	Disease duration (years)	CRP	Medication		DAS28	Clinical response		HMGB1 protein		HMGB1mRNA
						MTX	Pred		DAS28	ACR	ES	CG	
1	0	F	55	18	107	17.5	5 *	6.71			1.5	1	
	9				38			6.34	No	0	2	1.5	
2	0	F	52	6	14	7.5	7.5	6.00			1.5	1.5	
	9				15			4.43	Mod	20	1	0.5	0
3	0	F	58	2	46	10		7.91			3	4	
	9				25			5.41	Mod	50	1	1.5	0
4	0	F	57	4	7	10		4.94			2	2	
	9				34			4.41	No	20	2	2	Up
5	0	M	56	7	44	15		5.95			2	2.5	
	9				7			2.90	G	70	3.5	3	
6	0	F	69	1	69	15	5	7.39			2	3	
	9				7			1.79	G	70	1.5	2.5	Up
7	0	F	66	14	34	10		5.64			2	2	
	9				16			4.73	Mod	0	1	0.5	Down
8	0	F	66	10	18	12.5		4.83			3.5	4	
	9				19			4.23	Mod	0	0.5	2	Down
9	0	M	25	0.6	26	17.5	7.5	5.62			.5	3	
	9				25			3.91	Mod	20	3	4	

* Patient 1 also received 150 mg/day cyclosporine before and during the study period.Pat = Patient, W = week, CRP = C-reactive protein, MTX = Methotrexate (mg/week), Pred = Prednisone (mg/day), DAS28 = disease activity score calculated on 28 joints, ACR = American College of Rheumatology, HMGB1 = High-mobility group box chromosomal protein 1, ES = Erik Sundberg, CG = Cecilia Grundtman, F = Female, M = Male, No = Non, Mod = Moderate, G = Good.

In repeated biopsies from each patient before and after infliximab treatment, expression of CD markers for T-cells (CD3), macrophages (CD68) and activated macrophages (CD163) were analysed. CD3 and CD163 were unaffected, whereas the number of CD68-positive cells decreased as a consequence of infliximab therapy (data not shown).

### Synovial HMGB1 protein expression not influenced by infliximab therapy

**Figure 1:**
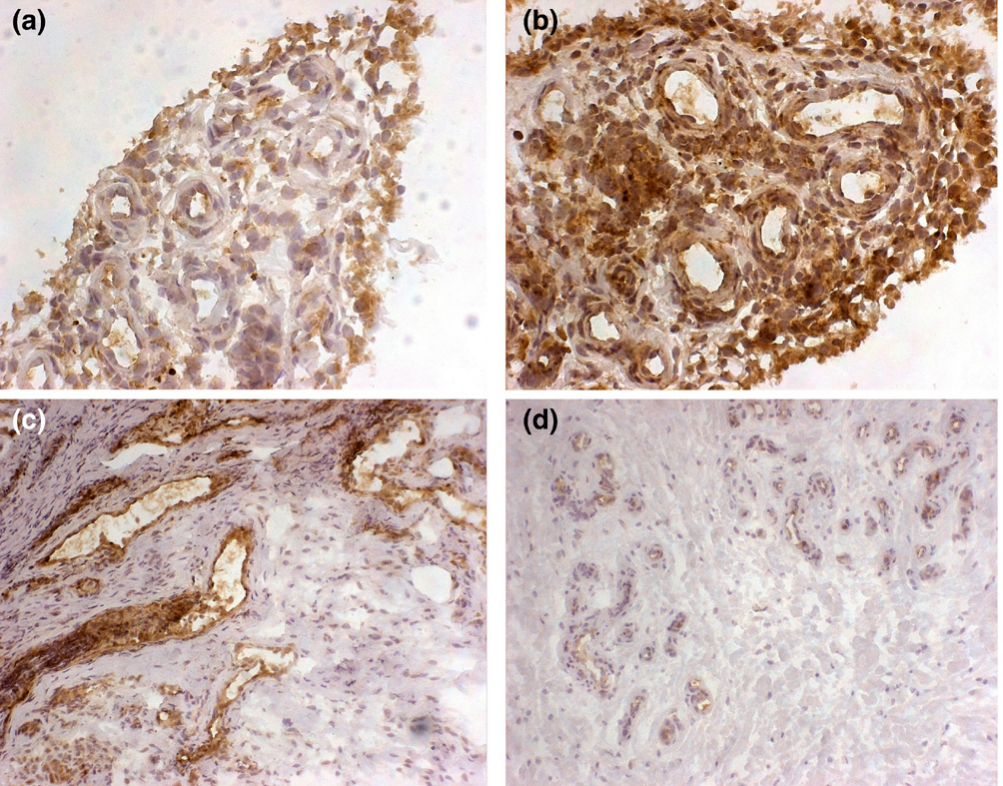
HMGB1 synovial protein expression before and during infliximab therapy as detected by immunohistochemistry. Nuclear, cytoplasmic and extracellular HMGB1 protein expression is evident in the RA synovia in the lining layer as well as in cellular infiltrates and endothelium. **(a) **Moderate aberrant synovial HMGB1 expression before the start of infliximab therapy from patient number 5. **(b) **The second biopsy in patient number 5 taken during infliximab treatment showing increased extranuclear HMGB1 staining. **(c) **A marked endothelial expression of HMGB1 is evident before infliximab therapy in synovitis obtained from patient number 7. **(d) **Infliximab therapy for nine weeks resulted in reduced endothelial HMGB1 expression in patient number 7. Original magnification ×250 for (a) and (b), ×100 for (c) and (d).

Aberrant synovial HMGB1 staining was evident in all nine patients before and during infliximab treatment and was most prominent in the lining layer, in areas with cellular infiltrates and in certain blood vessel walls. Both an increased (Figures 1a,b) and decreased (Figures 1c,d) presence of HMGB1 protein in the synovia during therapy could be detected, but there was no correlation with the clinical course of individual patients. In the group of six patients who responded to therapy, three had a decreased, two had an increased and one had an unchanged level of protein expression of HMGB1.

**Figure 2:**
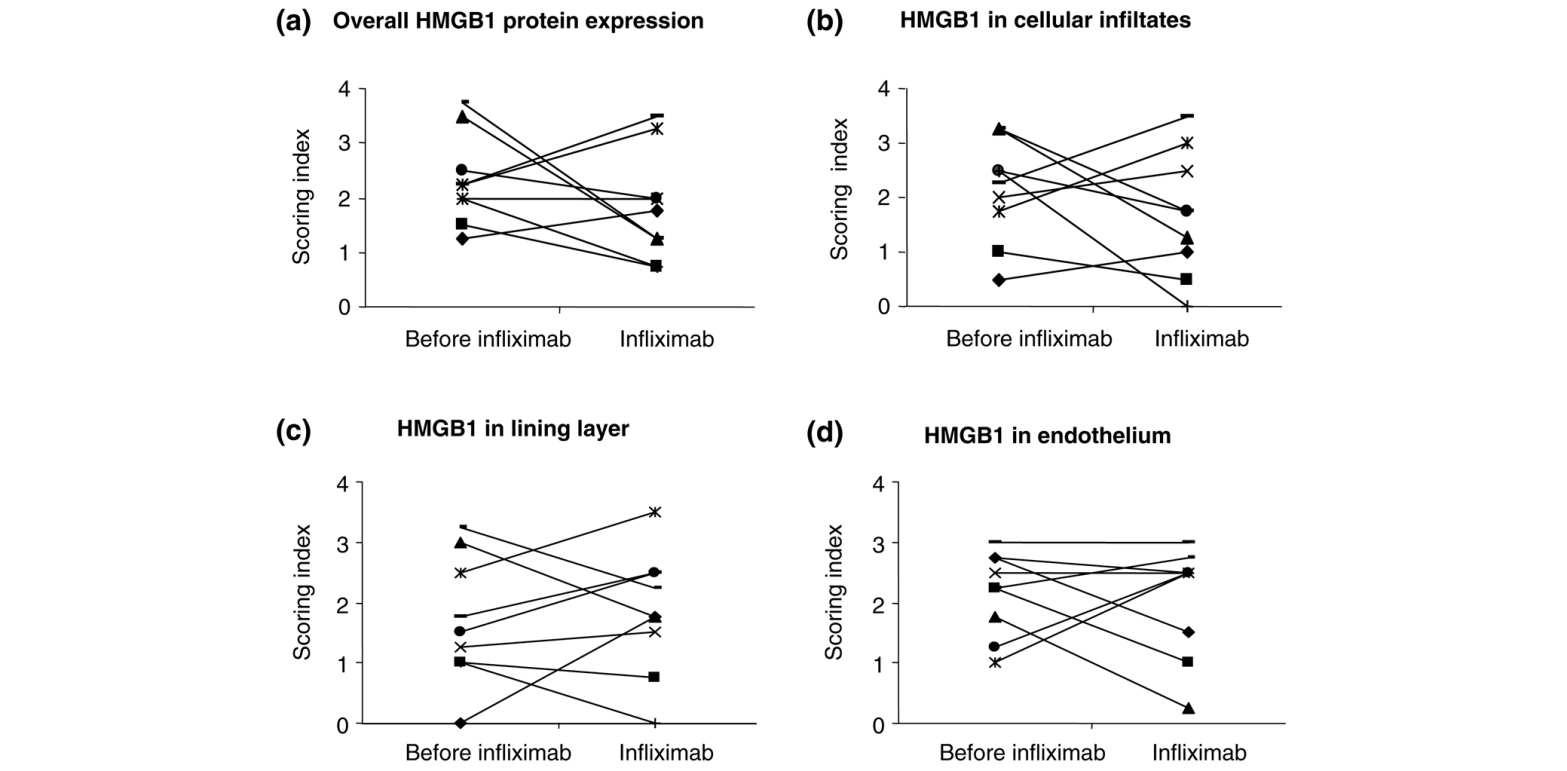
Change of HMGB1 synovial protein expression before and during infliximab therapy. Immunohistochemical scoring of HMGB1 protein expression was unchanged during the nine weeks of infliximab therapy. Values represent mean scores of highly concordant scoring recorded by the two independent investigators (ES and CG). The correlation was statistically confirmed by Spearman rank sum tests with a correlation coefficient (r_s_)of 0.74 (significant at p < 0.002). Scoring for each section was evaluated using a 0 to 4 scale with increments of 0.5. Index 0 corresponds to no HMGB1 expression and 4 to highest degree of HMGB1 protein expression. Separate analyses were performed for lining layer, vessels and cellular infiltrates in each of the sections. **(a) **Change of overall HMGB1 protein expression for each patient. **(b) **Change of HMGB1 expression in cellular infiltrates. **(c) **Change of HMGB1 expression in lining layer. **(d) **Change of HMGB1 expression in endothelium.

There was no significant difference in the group in the overall HMGB1 protein distribution before or after infliximab therapy (Figure 2). Neither were any consistent changes (nuclear, cytoplasmic or extracellular) recorded for HMGB1 expression when different biopsy compartments, including lining layer, cell infiltrates and endothelium, were analysed separately.

### HMGB1 mRNA expression not influenced by infliximab therapy

**Figure 3:**
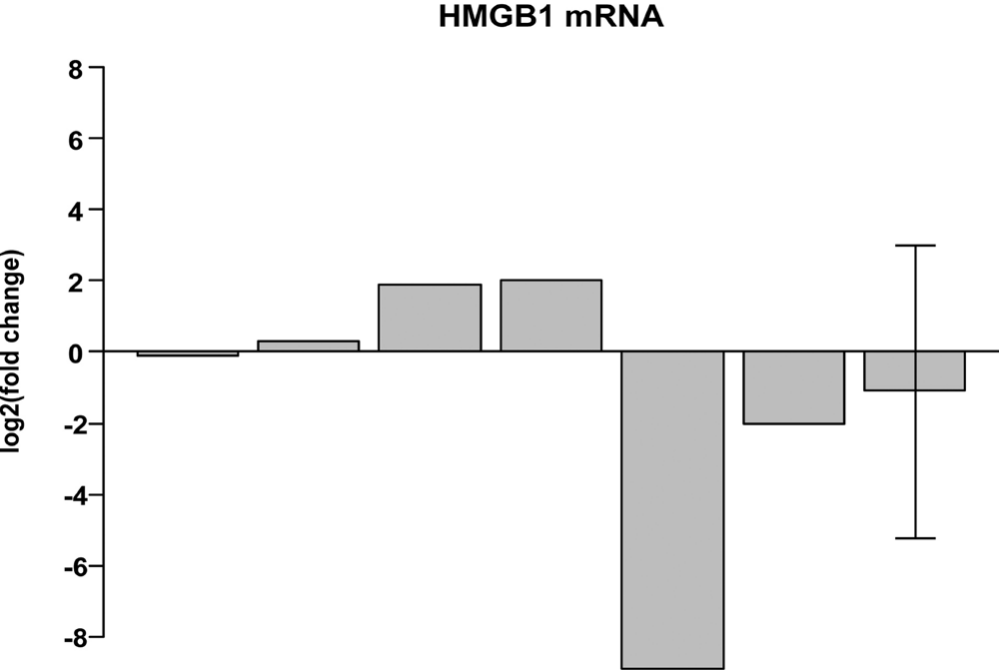
Change of HMGB1 synovial mRNA expression before and after infliximab therapy. HMGB1 mRNA expression as determined by reverse-transcriptase PCR for six patients. Values are expressed as fold-change of up- or down-regulation using a log_2_-scale. The last bar to the right represents the average value for the whole group and the standard deviation is indicated by whiskers.

In the group of six patients studied by RT-PCR two individuals had increased, two had decreased and two patients had unchanged HMGB1 mRNA levels, with no correlation to clinical outcomes. Taken together, the results indicated no significant change of HMGB1 mRNA as a consequence of infliximab therapy (Figure 3).

## Discussion

To the authors' knowledge, this is the first clinical study examining the effect of TNF blockade on synovial HMGB1 expression. The expression of synovial HMGB1 protein and mRNA in RA synovitis remained unaffected by TNF blockade for nine weeks and there was no correlation with the clinical course of arthritis. This was somewhat surprising as the original discovery of extranuclear and extracellular HMGB1 translocation was derived from studies of macrophages stimulated by TNF, IL-1β or endotoxin. Blocking intra-articular TNF by infliximab might therefore theoretically inhibit synovial HMGB1 release. However, this could not be verified from the results of the present study. There are several additional molecules such as IL-1β, IFN-γ, IFN-β, Nitric oxide and multiple TLR ligands that are potent promotors of HMGB1 translocation and extracellular release, and expression of all these mediators may possibly remain unchanged during infliximab therapy [43-45]. Supporting the clinical data for the TNF-independent release of HMGB1 are results obtained using a sensitive HMGB1 Elispot assay, which demonstrated that TNF has a distinctly inferior capacity to stimulate HMGB1 release from cultured macrophages compared with other molecules such as endotoxin, IFN-γ [46] or IL-1β (authors' unpublished data).

A similar arthroscopic-guided tissue sampling method and immunohistochemical analysis have previously been used to study the therapeutic effects of systemic infliximab therapy on the synovial expression of other important pro-inflammatory molecules [38,47]. Infliximab treatment in another small cohort of patients with RA saw the number of TNF-synthesising cells readily reduced and the production of IL-1α and IL-1β inconsistently down-regulated[47]. IL-15 was not inhibited by anti-TNF therapy in the same cohort of patients with RA as evaluated in the present study [38]. Therefore, beneficial clinical results after infliximab therapy may occur despite the fact that active cytokines such as IL-1, IL-15 and HMGB1 prevail during synovitis. Using a similar methodology contrasting results regarding HMGB1 expression in RA synovitis based on therapy with intra-articular corticosteroid injections was recently demonstrated [48]. Local intra-articular corticosteroid treatment clearly down-regulated the aberrant extranuclear expression of HMGB1. Likewise, TNF and IL-1β, but not IL-1α, were strongly suppressed by intra-articular corticosteroid therapy.

In the present study HMGB1 was analysed in a semi-quantitative way using conventional manual microscopy. Computerised image analysis was not an option because this technology does not enable discrimination between the constitutive nuclear HMGB1 expression and the aberrant presence of cytoplasmic and extracellular HMGB1 causing pathology.

The question regarding a functional relationship between TNF and HMGB1 in rheumatoid synovitis cannot be fully clarified in the present study due to the relatively limited number of patients with RA. However, it is proposed that the results can be interpreted in at least two different ways: HMGB1 and TNF could either act independently of each other; or HMGB1 could act upstream of TNF in the pro-inflammatory cascade. The latter explanation is favoured, since it has previously been reported that HMGB1 is a potent inducer of TNF production in cultured macrophages and dendritic cells [16,49,50]. In addition, it has recently been demonstrated that HMGB1 up-regulates TNF transcription by direct binding to the TNF promotor in osteoclasts [51]. Furthermore, successful HMGB1-targeted therapies in experimental sepsis, arthritis and brain ischaemia strongly down-regulate *in vivo *synthesis of TNF [29,52,53]. Another possibility to explain the lack of correlation between the clinical course and HMGB1 expression following infliximab treatment could be that HMGB1 may be more dependent on the IL-1 pathway than the TNF pathway in the pathogenesis of arthritis. This theory is also supported by the fact that intra-articular HMGB1 injections cause arthritis in wild-type mice but not in IL-1 type I-receptor deficient mice [5].

Considering that exaggerated and dysregulated HMGB1 release may lead to aggravated inflammation and tissue destruction in chronic arthritis, the results of the present study suggest that HMGB1 may serve as a possible TNF-independent target molecule for biological therapy. Future work is required to elucidate the potential of this strategy.

## Conclusion

A number of published observations have demonstrated that the pro-inflammatory mediator HMGB1 has a functional impact on the pathogenesis of arthritis. The results presented here reveal an unaffected expression of HMGB1 at protein and mRNA levels in synovia obtained from patients with RA from repeated biopsies before and during well-established TNF blockade with infliximab. These results are interpreted as an indication of TNF-independent HMGB1 expression with subsequent residual HMGB1 biological activity in RA synovitis in spite of infliximab therapy. It is concluded that these findings support attempts to generate novel HMGB1-targeted therapies in chronic arthritis.

## Abbreviations

ACR = American College of Rheumatology; DAS28 = Disease Activity Score calculated on 28 joints; HMGB1 = igh-mobility group box chromosomal protein 1; IFN = Interferon; IL = Interleukinl; RA = Rheumatoid arthritis; RAGE = Receptor for advanced glycated end-products; mAb = Monoclonal antibodies; RT-PCR = Reverse-transcriptase PCR; soluable RAGE = sRAGE; TLR = Toll-like receptor; TNF = Tumour necrosis factor.

## Competing interests

The authors declare that they have no competing interests.

## Authors' contributions

UA, HEH, A-KU, ES and CG designed the study. EaK performed the arthrosopies and synovial sampling. SE collected clinical patient data. ES and CG performed the immunohistochemical stainings and the immunohistochemical analysis. JL performed the RT-PCR and mRNA analysis. ES, CG, UA and HEH prepared the manuscript. All authors read and approved the final manuscript.
